# Maiden immunization coverage survey in the republic of South Sudan: a cross-sectional study providing baselines for future performance measurement

**DOI:** 10.11604/pamj.2013.16.110.3164

**Published:** 2013-11-23

**Authors:** William Mbabazi, Anthony K Lako, Daniel Ngemera, Richard Laku, Mostafah Yehia, Nathan Nshakira

**Affiliations:** 1 USAID/SPS Project in South Sudan, Management Sciences for Health, 784 Memorial Dr Cambridge, MA 02139, United States; 2 American Red Cross International Programs, P.O. Box 41275-00100 Nairobi, Kenya; 3 Ministry of Health, P.O. BOX 5 Juba, Republic of South Sudan; 4 UNICEF South Sudan Country Office, P.O. BOX 45 Juba, Republic of South Sudan; 5 WHO/South Sudan Country Office, P.O Box 5 Juba, Republic of South Sudan

**Keywords:** Immunization Coverage Survey, South Sudan

## Abstract

**Introduction:**

Since the comprehensive peace agreement was signed in 2005, institutionalization of immunization services in South Sudan remained a priority. Routine administrative reporting systems were established and showed that national coverage rates for DTP-3 rose from 20% in 2002 to 80% in 2011. This survey was conducted as part of an overall review of progress in implementation of the first EPI Multi-Year Plan for South Sudan 2007-2011. This report provides maiden community coverage estimates for immunization.

**Methods:**

A cross sectional community survey was conducted between January and May 2012. Ten cluster surveys were conducted to generate state-specific coverage estimates. The WHO 30x7 cluster sampling method was employed. Data was collected using pre-tested, interviewer guided, structured questionnaires through house to house visits.

**Results:**

The fully immunized children were 7.3%. Coverage for specific antigens were; BCG (28.3%), DTP-1(25.9%), DTP-3 (22.0%), Measles (16.8%). The drop-out rate between the first and third doses of DTP was 21.3%. Immunization coverage estimates based on card and history were higher, at 45.7% for DTP-3, 45.8% for MCV and 32.2% for full immunization. Majority of immunizations (80.8%) were received at health facilities compared to community service points (19.2%). The major reason for missed immunizations was inadequate information (41.1%).

**Conclusion:**

The proportion of card-verified, fully vaccinated among children aged 12-23 months is very low at 7.3%. Future efforts to improve vaccination quality and coverage should prioritize training of vaccinators and program communication to levels equivalent or higher than investments in EPI cold chain systems since 2007.

## Introduction

Since the initiation of Expanded Programme on Immunization in 1974 by the World Health Organization (WHO)[1, 2], there has been substantial increases in the vaccination coverage against the six major vaccine-preventable childhood diseases: tuberculosis, polio, diphtheria, pertussis, tetanus and measles. In countries where accurate recording of immunization and reporting of diseases is well established, most vaccine-preventable diseases are at or near record lows [3].

Worldwide, vaccination coverage (measured by estimates of the third dose of Diphtheria Tetanus and Pertussis vaccine – DTP-3) has been on an upward trend; increasing from 74% in 2000 to a record 85% in 2010 [4]. As of 2010, a total of 149 countries had attained or were on track to achieve the 90% coverage goal for DTP-3 [4]. Most WHO regions sustained the positive DTP-3 coverage trends even after surpassing the 80% mark. The African region reached a record high vaccination coverage level of 74%.[5, 6] However, the good coverage levels should not be used to mask the high absolute numbers of infants who continue to miss their 3rd DTP dose; estimated to have been 22.4 million in 2011[5].

In South Sudan, a nationwide program for immunization was established in 2005 following the signing of the Comprehensive Peace Agreement (CPA) in the same year. In 2006, the Government of Southern Sudan formulated the “Health Policy, 2007-2011” and the “Basic Package of Health and Nutrition Services”[7, 8]. Based on the two broad policy guiding documents of the Ministry of Health, the EPI program with support from the partners prepared a “2007-2011 Comprehensive Multi-Year Plan (cMYP) for Immunization” [9]. This plan formed the basis for immunization systems investment and implementation until July 2011 when the country gained independence.

A comprehensive program review was conducted in 2011 to assess progress and the current context and inform the development of a second generation multi-year plan for immunization (2012-2016). The administrative reporting system had documented an increase in DTP-3 coverage from 20% in 2007 to 80% in 2011. In the same period, the DTP-1 to DTP-3 dropout rate was documented to have reduced from 41% in 2007 to 26% in 2011. There were wide disparities in DTP-3 coverage between the states and in each state, between the counties [10].

As part of the comprehensive review process, this study sought to determine baseline immunization coverage indicators that would be used to set benchmarks for the 2012-2016 multi-year plan for immunization. This survey would also identify specific factors for vaccination failure in order to inform evidence-based strategies/interventions for raising immunization coverage. In a newly formed Republic of South Sudan, running on a transitional constitution [11], a revised health sector development plan for 2011-2015 [12] and a newly elected democratic government, it was important to provide coverage rates against which future immunization program performance measurements will be done. South Sudan lacks dependable census figures and large population movements that occurred over the CPA period make it difficult to estimate actual or changes in immunization coverage.

## Methods

**Study site** The Republic of South Sudan (RSS) is the youngest nation in the world. It lies between latitudes 3o and 13oN, and longitudes 24o and 36oE. It is covered in tropical forest, swamps, and grassland. South Sudan covers an estimated area of 640,000 km2, of which 18% consists of White Nile and its related tributaries and swamps. At the time of the reported survey, RSS was administratively divided into 10 States and 79 counties. The counties are further divided into 514 Payams, 2,159 Bomas and 26,544 major villages [8].

According to a census carried out in April 2008, the population of South Sudan was 8,260,490, a figure that was projected to rise to 9,480,000 by January 2009 [8, 13]. The census population estimates were disputed both locally and internationally. The annual population growth rate of 3% is given by the National Bureau of Statistics (NBS) to project the annual populations after 2008. The majority (83%) of the population lives in rural areas [13].

South Sudan is one of the poorest countries in the world with about half of its population (51%) living on less than 1 US$ per day. The economy of South Sudan is one of the world′s weakest and most underdeveloped; having little existing infrastructure and the highest maternal mortality and female illiteracy rates in the world. The vast majority of the population is engaged in rural subsistence farming and cattle herding. Living conditions are deprived; with poor access to potable drinking water (less than 50%), poor access to proper sanitation (less than 7%) and high illiteracy rates among adult population (88% among women and 63% men) [14].

The provision of health services in South Sudan is far from adequate. As a result, basic health indices are still very poor. Data from household health survey indicates that maternal mortality ratio was 2,054 per 100,000 live births (in 2006), infant mortality rate was 84 per 1,000 live births in 2010 and under-five mortality was 106 per 1,000 live births (in 2010) [15, 16]. The 2006 and 2010 household health surveys also indicated that card verified DTP-3 coverage had declined from 10.3% to 5.4% respectively. In the two surveys, the proportion of fully immunized declined from 9.4% to 4.3%, despite contrary trends from the administrative reporting system. The 2010 survey report did not provide qualitative explanation for the declining trends in immunization performance indicators.

### Study design

A cross-sectional community-based study was conducted using the WHO (30X7) EPI cluster sampling survey methodology, as described in the 1991 WHO manual titled “Immunization Coverage Cluster Survey – Reference Manual” [17, 18]. The survey was conducted in all 10 states of RSS; each with a sample of 30 clusters to give state-specific coverage estimates. Another 30 clusters were selected at national level, to enable rapid estimation of national coverage needed for new vaccine introduction application to the Global Alliance for Vaccines and Immunization (GAVI) in August 2012. A total of 330 clusters were thus surveyed but this report presents weighted analysis of the 300 clusters collected from 10 administrative states of the country.

Data was collected from mothers or caretakers of children aged 12-23 months in the selected households using pre-tested, semi-structured questionnaires. Information collected included the socio-demographic characteristics, immunization status of the children and factors influencing vaccination utilization. Where available, Child Health Cards were used to assess the immunization status of the children. In the absence of the child health cards, mothers/caretakers of the respective children were asked to recall the immunization history, guided by the count of vaccinations received in each site as provided for in the national schedule. BCG Coverage was verified by physically checking the vaccination site of all surveyed children for the presence of a BCG scar.

### Ethical approval

Approval was obtained from the South Sudan Ethics Review Committee of the Ministry of Health. Permission to conduct the state-specific surveys was also sought from the State Ministries of Health. Informed consent was sought from the study participants before administration of the questionnaire.

### Data management and analysis

The quantitative data from the field was double entered into a database designed using Epi Info™ 3.5.3. The database was then imported into SPSS for analysis. Univariate analysis was done to determine immunization performance indicators.

The outcome measures of the study included weighted immunization coverage estimates at national level derived from the 10 state-specific surveys by antigens described in the national schedule. Other weighted analysis outcomes included the proportion of card verified fully immunized children, proportion of children who had received none or part of the vaccines on the immunization schedule, card retention rates and dropout rates. Maternal and child health indicators on malaria, diarrhoea, antenatal care, Polio eradication program and nutrition support services were measured but are not presented in this article

### Limitations

This study was prone to recall bias as the respondents, who did not have the child health cards, were asked to recall the vaccines that were administered to their children, 1 -11 months earlier. Some of the respondents did not give specific reasons for not taking their children for the missed vaccination.

## Results

### General characteristics of the survey respondents

A total of 10,727 households were visited in the 300 sampled clusters surveyed in 10 states; in which caretakers for 2,246 children aged 12-23 months were interviewed. Majority of the respondents (73.5%) had no formal education; 66.6% with no education at all, and 6.9% literate but with no formal schooling. The proportion of respondents who had attained at-least a Primary School education was 26.5%; with only 5.8% having attained post primary education. Majority of participants (87.5%) reported to have been residents of their survey location for more than 2 years compared to 12.5% that had residence status of less than 2 years. Only 17.6% of the children in the survey were ranked first in birth order. Second, third, fourth and fifth birth orders were 20.7%, 19.8%, 15.4% and 10.6% respectively. The rest of the children (15.9%) were of birth order sixth and above.

### The National immunization indicators for South Sudan

Of the 2,246 children surveyed, 1,475 (65.7%) had received child health cards. However, only 51.0% (752 of 1,475) who received the cards had them shown to the survey teams on the survey date. Table 1 below presents the immunization coverage as verified from the cards seen, and based on recall by the interviewed caretakers (where no cards were available).

**Table 1:** Immunization coverage in surveyed children 12-23 months of South Sudan: Jan-Apr 2012

Antigen	Valid coverage… (%) before 1^st^ birthday	Crude Coverage (95% Confidence Interval)		Crude Coverage as of survey date (All received doses)
		Card only (All Doses) before 1^st^birthday	Card + History (before 1^st^ birthday)	Card + History
BCG	28.3	29.9 (21.3 – 38.5)	71 (62.5–79.5)	75.4
DPT1	25.9	31.4 (22.7– 40.1)	73.0 (64.6 – 81.4)	79.1
OPV1	23.7	29.3 (20.7 – 37.9)	73.1 (64.8 – 81.4)	79.7
DPT3	22.0	24.3 (16.2 – 32.4)	45.7 (36.3 – 55.1)	55.4
OPV3	22.8	24.4 (16.3 – 32.5)	46.0 (36.6 – 55.4)	58.5
MCV	16.8	22.9 (15.0 – 30.8)	45.8 (36.4 – 55.2)	62.4
Fully immunized	7.3	20.2 (12.6 – 27.8)	32.2 (23.4 – 41.0)	47.4
BCG-MCV dropout rate	39.6	22.0	34.9	18.2
DPT1-DPT3 dropout rate	21.3	23.6	38.4	30.9

… Valid coverage is based on doses whose dates were recorded in CHC, and were administered according to the minimum intervals recommended in the national immunization schedule

This study documents that 164 (7.3%) of the children 12-23 months had card verified full immunization with antigens given on schedule. This figure increased to 20.2% (n = 454) when the filter of doses received on schedule was removed. The full immunization coverage by card and history (crude coverage before 1 year) was 32.2%.

As of the survey date, the crude coverage (card plus history) by antigen was determined as: BCG (75.4%), OPV1 (79.7%), OPV3 (58.5%), DTP-1(79.1%), DTP3 (55.4%), measles (62.4%). The drop-out rate between the first and third dose of DTP was 30.9%. When these rates were filtered for immunizations received before 1st birth day, the crude coverage by antigen reduced to: BCG (71.0%), OPV1 (73.1%), OPV3 (46.0%), DTP-1(73.0%), DTP3 (45.7%), measles (45.8%). The drop-out rate between the first and third dose of DTP then increased to 38.4%.

The surveyed crude coverage rates (before 1 year) were compared with the corresponding administrative reporting period of 2010 (WHO/UNICEF JRF), and found to be all lower; as presented in Table 2.

**Table 2:** Coverage survey rates compared to 2010 Administrative reporting period

Antigen	Survey crude coverage (Before first birthday)	Survey coverage range (95% Confidence interval)	Administrative Coverage (2010 comparative period)
BCG	71.0	62.5–79.5	91.0
DPT1	73.0	64.6 – 81.4	97.0
DPT3	24.3	16.2 – 32.4	71.0
OPV3	24.4	16.3 – 32.5	71.0
MCV	22.9	15.0 – 30.8	106.0

Four out of every five vaccinations received by surveyed children (80.8%) were received from health facilities; while the remaining 19.2% were from community service delivery points.

A total of 1,180 mothers/caretakers for surveyed children that were not fully immunized (as at survey date) were asked about the main reasons why the children had not received the scheduled immunizations. Nearly all of these respondents (1,157; 98.1%) gave reasons for missed immunizations, presented in Table 3 below. Seventy three percent of the respondents who gave reasons indicated more than one reason.

**Table 3:** Specific reasons given for missed immunizations among children 12-23 months (N = 1,180)

Category/reason	Percent	Category/reason	Percent
**Lack of information:**	**41.1**	**Family obstacles:**	**30.0**
Unaware of need for immunization	18.6	Mother too busy	15.7
Unaware of need for multiple doses	14.6	Household poverty	6.4
Place/time of immunization not known	11.2	Ill-health	9.9
Fear of side-effects	6.6	Family refused	3.9
Wrong ideas about contra-indications	3.1		
**Obstacles in system:**	**36.7**	**Lack of motivation:**	**23.1**
Immunization station too far	21.2	Postponed until another time	10.8
Long waiting time	3.7	No faith in immunization value	11.3
Vaccine out of stock	4.6	Encouraged not to go	3.5
Vaccinator absent	5.2		
Time of service not convenient	5.5		
Vaccinator rude/ not adequately trained	3.2		
Child ill (brought; not immunized)	2.2		
Charges for EPI services; not right	1.4		

Multiple reasons within and beyond each of the four categories were given by a number of respondents

This survey showed that there are variations in coverage between the 10 administrative states (Table 4). The highest and lowest valid DTP-3 coverage were found in Central Equatoria (39.6%) and 3.1% in Lakes state.

**Table 4:** State-specific immunization coverage surveys in South Sudan; Jan-Apr 2012

State	Households Visited	Children 12-23 months surveyed	No (%) of children with Child Health Cards	Percentage of children with Valid DTP-3 doses (Card only before 1yr)	Crude DTP-3 coverage before 1^st^ Birthday (Card + History)	Crude DTP-3 coverage as of Survey Date (Card + History)
Central Equatoria	1,222	225	152 (67.6)	39.6	53.6	73.0
Eastern Equatoria	1,253	225	183 (81.2)	24.0	31.3	36.9
Jonglei	1,280	210	97 (46.3)	28.6	62.8	69.5
Lakes	941	224	59 (26.3)	3.1	15.8	20.4
Northern Bahr El Ghazal	1,020	222	128 (57.6)	20.0	44.3	54.3
Unity	1,415	227	111 (49.0)	24.2	44.1	52.4
Upper Nile	887	218	102 (46.6)	17.0	47.7	59.2
Warrap	1,079	229	27 (12.0)	5.7	39.3	52.0
Western Bahr El Ghazal	768	231	145 (62.6)	22.6	57.7	62.8
Western Equatoria	862	235	153 (65.1)	27.9	52.8	58.4
**National Estimate**	**10,727**	**2,246**	**1157 (50.5)**	**22.0**	**45.7**	**55.4**

## Discussion

The educational attainment of surveyed mothers/caretakers, at 73.5% with no formal education; 20.7% with primary education and 5.8% post-primary education, compares closely with samples from other surveys conducted in South Sudan in recent years. The 2006 household survey sample had 64 percent of mothers of children 0-59 months with no formal education; and the 2009 malaria indicators survey found this proportion at 73 percent [14–16, 19].

The birth order of surveyed children suggests high fertility levels in the surveyed communities. This usually translates into a large burden of child care on the mothers, on top of their other household and gender related responsibilities. As has been documented in Bangladesh, India and Guatemala, having more children may cause resource constraints, which have a negative effect on healthcare utilization, including immunization [20–22].

This study found that the fully immunized coverage (card + history as at survey date) for South Sudan was 47.5%. However, this rate significantly reduced to 32.2% (CI of 30.2 – 34.2) when filtering was done for immunizations received before the first year of age, where the most benefits are expected. The rate for fully immunized coverage before first birthday based on card records is even lower, at only 20.2%; a reflection of incomplete provision of cards, and inadequate card retention. When screened for adherence to the schedule time and interval between doses, the fully immunized coverage reduced further to 7.3%.

Comparison of valid and crude coverage by card revealed high level of invalid doses, especially with respect to OPV0, MCV, OPV1 and DPT1. High rates of untimely vaccinations have been reported in other study settings [23, 24]. The implication of delay in receipt of vaccines is that a pool of children with incomplete or no immunization quickly builds up. High rates of untimely vaccinations reflect inadequate discussion about the immunization schedule during the EPI service delivery process; and limited recording of dates for immunization in the immunization register and on child health cards. Studies on the subject have shown that untimely vaccinations may be attributed to limited skills and confidence among health workers, inadequate attention to EPI data management, pressure to deliver services quickly when overwhelmed by turn-up, and inadequate mentoring and monitoring of immunizations by supervisors [25]. These factors need to be prioritized in immunization quality and coverage improvement planning for South Sudan.

The drop in coverage when the first birth date is used to filter is across all antigens in the national immunization schedule. The rates attained (all antigens) at survey date are outside the upper end of the confidence interval of the rates at first birthday, implying that the increase between the two rates (before and after one year) is statistically significant. This means that some of the children not reached with immunization in the first year of life are getting the opportunity to start and complete immunizations in the second year.

Immunization of children above one year should be looked at in the context of South Sudan and should indeed be promoted. Although the benefits of childhood immunization are highest in the first year of life; children that are not (or are inadequately) immunized continue to be at risk of illness; death and long-term disability due to vaccine preventable diseases until five years of age and even older.[26] Therefore, vaccination of children above 1 year should continue, including starting the schedule in the second year, while targeted behavior change communication for early start of immunizations is promoted at community levels.

MCV coverage is still low in RSS, with 62% of surveyed children immunized by the time of survey, and only 46% before the first birthday. Card-verified MCV vaccination was only 23%, with only 17% verified as valid (received after 39 weeks of age). Such low MCV coverage means that nearly 80% of each annual birth cohort is not vaccinated and in turn partially explains the frequent occurrence of measles outbreaks that continue to be experienced in many parts of the country.

The under-one year crude (card and history) coverage rates from this survey, when compared to the administrative reported rates for infants less than 1 year old in South Sudan for 2010, are lower for all antigens. The survey cohort of children 12-23 months old corresponds closely to the children targeted for immunization before the first birthday in 2010. The lower survey coverage reflects over-estimation of coverage by the administrative reporting system. Coverage over-estimations in the administrative system in South Sudan are especially attributable to under-estimation of the denominators for the total number of infants below 1 year based on the 2008 census projections. The unknown returning populations into the country, that is never been factored into the national denominator estimations only adds to the problem.

Over-reporting of infant immunization rates could also be a factor of: a) age misclassification of immunization recipients, b) erroneous tallying of immunizations, c) weak data quality assurance mechanisms in the EPI information management system and d) deliberate presentation of all children immunized as infants where the reporting formats had no provisions for reporting of children above 1 year old. Mechanisms need to be instituted to ensure and regularly audit EPI data quality, including efforts to address data quality and data utilization during in-service training and supportive supervision visits.

Four out of every five vaccinations received by surveyed children (80.8%) were from health facility service delivery points; while the remaining 19.2% were from community service points. Immunizations received from health facilities in South Sudan should not be interpreted as static services because many health facilities do not have permanent cold chain equipment to function as a fixed immunization service delivery point. Secondly, it is a deliberate policy of the national immunization program to promote integration of EPI services with primary health structures by supporting outreach sites in health facilities that do not yet have immunization equipment. In the same tone, the reported 19 percent from community service delivery points should not be interpreted as outreaches and thus under-estimate the importance of this service delivery strategy. This is because there are several outreaches and/or mobile immunization clinics operated at health facilities without vaccine fridges. Whatever the case, this survey suggests that the country needs to invest more in cold chain expansion and establishment/re-opening of integrated outreach immunization services as a strategy for raising coverage, in line with the RED approach [27].

As in other countries, reasons for immunization failure were more related to lack of information and obstacles in the immunization system, cited by respondents with children who were not fully immunized in 68% and 64% cases respectively [28, 29]. This reflects inadequate communication and social mobilization specific to routine immunization; and limited inter-person communication during EPI service delivery. South Sudan should strengthen social mobilization for routine immunization, including specific communication to all health service users and community leaders to address the information needs identified in this study. Emphasis should be placed on enhancement of health worker skills in interpersonal communication to provide reliable and accurate information about immunization, an approach that has been demonstrated to be effective in A/H1N1 influenza vaccination uptake. [30] Targeting training schools to provide trainee health workers with immunization communication skills has been documented as an effective primary intervention [31]. Bivariate and multivariate analysis of state-specific immunization coverage surveys data is urgently needed to determine other factors relevant in communication and social mobilization in this new country [32, 33].

Similarly, actions to improve the immunization services system on the elements mentioned would increase utilization levels. Lastly, although obstacles in the family and lack of motivation were given by a smaller proportion of the respondents; at 44% and 37% respectively, the program needs to map and involve the multi-sectoral players needed to address the constraints to utilization of immunization services at individual, family and community level.

## Conclusion

The utilization of immunization services in South Sudan, could be rated as very low. However, in the context that this is a country emerging out of decades of civil war, these results show that high valid coverage could be attained with provision and promotion of child health card retentions, training of vaccinators on adherence to the vaccination schedule and mass education on the importance of timely receipt of routine infant immunization doses. There is need to strengthen communication, education and information skills of health workers to improve immunization service provision and health education to mothers/guardians.
